# Development of a Polymicrobial Checkerboard Assay as a Tool for Determining Combinatorial Antibiotic Effectiveness in Polymicrobial Communities

**DOI:** 10.3390/antibiotics12071207

**Published:** 2023-07-20

**Authors:** Caroline Black, Hafij Al Mahmud, Victoria Howle, Sabrina Wilson, Allie C. Smith, Catherine A. Wakeman

**Affiliations:** 1 Department of Biological Sciences, Texas Tech University, Lubbock, TX 79409, USA; caroline.black@ttu.edu (C.B.);; 2 Department of Mathematics and Statistics, Texas Tech University, Lubbock, TX 79409, USA; 3 Department of Honors Studies, Texas Tech University, Lubbock, TX 79409, USA

**Keywords:** polymicrobial, antibiotic susceptibility, bacterial communities

## Abstract

The checkerboard assay is a well-established tool used to determine the antimicrobial effects of two compounds in combination. Usually, data collected from the checkerboard assay use visible turbidity and optical density as a readout. While helpful in traditional checkerboard assays, these measurements become less useful in a polymicrobial context as they do not enable assessment of the drug effects on the individual members of the community. The methodology described herein allows for the determination of cell viability through selective and differential plating of each individual species in a community while retaining much of the high-throughput nature of a turbidity-based analysis and requiring no specialized equipment. This methodology further improves turbidity-based measurements by providing a distinction between bacteriostatic versus bactericidal concentrations of antibiotics. Herein, we use this method to demonstrate that the clinically used antibiotic combination of ceftazidime and gentamicin works synergistically against *Pseudomonas aeruginosa* in monoculture but antagonistically in a polymicrobial culture also containing *Acinetobacter baumannii*, *Staphylococcus aureus*, and *Enterococcus faecalis*, highlighting the fundamental importance of this methodology in improving clinical practices. We propose that this method could be implemented in clinical microbiology laboratories with minimal impact on the overall time for diagnosis.

## 1. Introduction

Antibiotic resistance is one of the biggest challenges faced when attempting to effectively treat patient infections. In certain cases, the initial treatment prescribed can mean the difference between a patient living or dying [1]. In some cases, antibiotic combination therapy, in which two or more antibiotics are prescribed, has been shown to help clear infections quickly and more effectively than standard mono-treatment [2,3]. Antibiotic combination therapy can be used if the infection requires a broader spectrum of therapy, if the infection is determined to be polymicrobial in nature, if antibiotic synergism is deemed helpful for treating the infection, or if the emergence of antibiotic resistance is seen while treating the infection [4]. Currently, the decision regarding which antibiotics to use when treating an infection is based on empiric and definitive antimicrobial therapy [5]. Empiric antimicrobial therapy is when an antibiotic (or antibiotics) is chosen by a clinician based on the presentation of symptoms when the patient first arrives at the hospital. This therapy usually consists of a broad-spectrum antibiotic designed to treat a variety of infections, since the infection-causing organism has not been speciated. This can have unfortunate consequences, such as killing beneficial gut microbiota [6]. Definitive antimicrobial therapy is when certain antibiotics are chosen after culturing microorganisms obtained from the infection site and determining the causative agent of disease. These antibiotics are typically more narrow-spectrum in nature and are designed to treat specific infections, but unfortunately, are not able to be given to the patient until the diagnosis is confirmed, leading to the loss of valuable time needed when combating infections effectively [5]. This delay in the prescription of narrow-spectrum antibiotics is due to the acknowledgment that early prescription without confirmation of diagnosis can result in treatment failure. Clinicians are also discouraged from prescribing narrow-spectrum antibiotics without diagnosis confirmation due to antimicrobial stewardship efforts to prevent over-prescription, leading to increased antibiotic resistance. There are current recommendations for antibiotic combination therapies for some species, such as vancomycin with an aminoglycoside and rifampin for methicillin-resistant *Staphylococcus aureus* [7], and ceftazidime and gentamicin (sometimes in combination with rifampin) for infections caused by *Pseudomonas aeruginosa* [8,9]. Combinations of antibiotics are chosen based on the assumption that synergism is conserved between species and strains of microorganisms. However, recent studies have shown that these interactions can change depending on both the species present and the antibiotics prescribed [10]. For example, the antibiotic combination of ciprofloxacin with metronidazole has been shown to allow for ciprofloxacin recalcitrance to develop in *P. aeruginosa* as the SOS response is induced [11]. Antibiotic tolerance can also be increased when certain species are present, such as when *P. aeruginosa* produces pyocyanin, allowing for increased antimicrobial tolerance for both itself and other species [12]. The microbial community plays a role in determining a species’ antimicrobial susceptibilities, and yet there is a paucity of research focusing on the community when determining antibiotic susceptibility [13]. Therefore, it is crucial to consider the community when prescribing antibiotics to treat infections effectively.

The checkerboard assay is one way of measuring the effects of antibiotic combination therapy. Varying concentrations of two antibiotics can be dispensed along the columns and rows to allow for the determination of minimum inhibitory concentration (MIC) for each antibiotic in combination [14]. The MICs are determined using visible turbidity readings or optical density [15]. Unfortunately, using the standard readout of visible turbidity and optical density readings from current checkerboard methods does not include the determination of viable cell numbers. While visible turbidity can demonstrate how a community affects the overall susceptibility of all species combined, it leaves the researcher unable to determine where the susceptibilities lie for individual species within the community. Another limitation of the visible turbidity assay is the sensitivity issue. First, dead cells can falsely contribute to turbidity. Second, live cell populations can be too dilute to be read via turbidity, masking the bacteriostatic versus bactericidal effects of certain drug treatments. The new methodology described herein allows for the determination of viable cell numbers via the use of colony-forming unit (CFU) counts as the readout for the checkerboard assay, thus allowing for the measurement of susceptibility changes for individual species in a polymicrobial community. No special laboratory equipment is needed, only selective/differential media for the species tested. This newly developed methodology also allows for a more accurate determination of antibiotic synergy versus antagonism by relying on equations that account for both visible turbidity and CFU counts. The addition of CFU counts to the calculations required to determine antibiotic synergy or antagonism allows for a more sensitive assay accurately reflecting the viable bacterial count post-antibiotic challenge. The addition of accurate CFU counts to the data obtained from the checkerboard assay is essential in helping one to understand exactly how antibiotic combination affects the clearing of pathogens known to cause infection when found in a polymicrobial community. As shown with our data, when CFU counts are obtained, even recommended antimicrobial combinations that work well on pathogens by themselves, such as gentamicin with ceftazidime for *P. aeruginosa*, can be antagonistic and counterproductive to patient treatment when a complex microbial community is present.

## 2. Results

**Figure 1:**
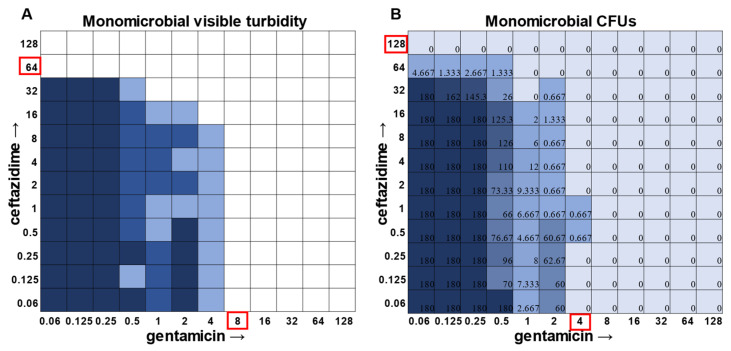
Turbidity versus CFU data in *Staphylococcus aureus* checkerboard assay. (**A**) Visible turbidity of *S. aureus* ATCC 29213 when grown in the monomicrobial condition. Data represent triplicate checkerboard assays with ceftazidime and gentamicin. Dark coloration indicates that turbidity was visible in the well in all replicates, medium coloration indicates that turbidity was visible in two replicates, and light coloration indicates that turbidity was visible in one replicate. Red boxes indicate determined MIC. (**B**) CFU data from the same triplicate plates with numbers representing the average of the triplicate CFUs detected. Wells displaying TNTC (too numerous to count) CFUs were assigned a number of 180 to enable the determination of averages across replicates. Red boxes indicate determined MIC.

**Figure 2:**
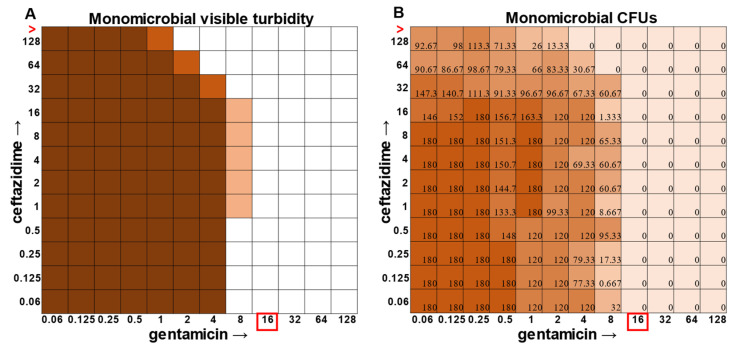
Turbidity versus CFU data in *Enterococcus faecalis* checkerboard assay. (**A**) Visible turbidity of *Enterococcus faecalis* ATCC 29212 when grown in the monomicrobial condition. Data represent triplicate checkerboard assays with ceftazidime and gentamicin. Dark coloration indicates that turbidity was visible in the well in all replicates, medium coloration indicates that turbidity was visible in two replicates, and light coloration indicates that turbidity was visible in one replicate. Red boxes indicate determined MIC. (**B**) CFU data from the same triplicate plates with numbers representing the average of the triplicate CFUs detected. Wells displaying TNTC (too numerous to count) CFUs were assigned a number of 180 to enable the determination of averages across replicates. Red boxes indicate determined MIC. Red greater-than sign indicates MIC > 128.

**Figure 3:**
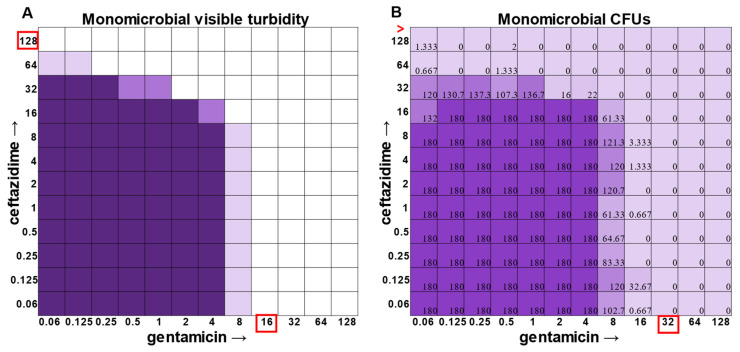
Turbidity versus CFU data in *Acinetobacter baumannii* checkerboard assay. (**A**) Visible turbidity of *Acinetobacter baumannii* ATCC 19606 when grown in the monomicrobial condition. Data represent triplicate checkerboard assays with ceftazidime and gentamicin. Dark coloration indicates that turbidity was visible in the well in all replicates, medium coloration indicates that turbidity was visible in two replicates, and light coloration indicates that turbidity was visible in one replicate. Red boxes indicate determined MIC. (**B**) CFU data from the same triplicate plates with numbers representing the average of the triplicate CFUs detected. Wells displaying TNTC (too numerous to count) CFUs were assigned a number of 180 to enable the determination of averages across replicates. Red boxes indicate determined MIC. Red greater-than sign indicates MIC > 128.

**Figure 4:**
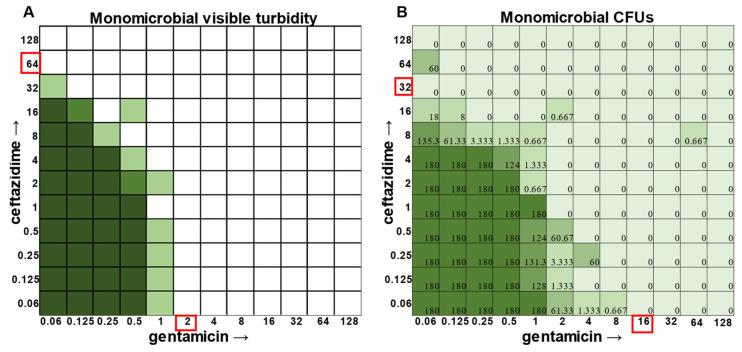
Turbidity versus CFU data in *Pseudomonas aeruginosa* checkerboard assay. (**A**) Visible turbidity of *Pseudomonas aeruginosa* ATCC 27853 when grown in the monomicrobial condition. Data represent triplicate checkerboard assays with ceftazidime and gentamicin. Dark coloration indicates that turbidity was visible in the well in all replicates, medium coloration indicates that turbidity was visible in two replicates, and light coloration indicates that turbidity was visible in one replicate. Red boxes indicate determined MIC. (**B**) CFU data from the same triplicate plates with numbers representing the average of the triplicate CFUs detected. Wells displaying TNTC (too numerous to count) CFUs were assigned a number of 180 to enable the determination of averages across replicates. Red boxes indicate determined MIC.

While visible turbidity observed via the human eye was comparable to OD_625_ readings (Supplementary Figure S5), when turbidity data were compared to the CFU data in the classic monomicrobial checkerboard setup, it became evident that viable cells could be detected in wells not displaying visible turbidity for all individual species tested (Figure 1, Figure 2, Figure 3 and Figure 4). Conversely, the presence of visible turbidity could also obscure the fact that the cells in particular wells are no longer viable. For example, *S. aureus* had detectable CFUs in 64 μg mL^−1^ ceftazidime, even though visible turbidity did not extend past 32 μg mL^−1^, whereas these same data showed that the presence of cells could sometimes be visibly detected in 4 μg mL^−1^ gentamicin, but these cells were no longer viable when plated (Figure 1). *E. faecalis* and *A. baumannii* also exhibited quantifiable viable cell counts where visible turbidity was lacking, although for the most part, the turbidity and CFU data were consistent with each other (Figure 2 and Figure 3). Even more strikingly, *P. aeruginosa* had a visible turbidity MIC of 2 μg mL^−1^ for gentamicin, and yet there were still large quantities of growth in wells containing a concentration of 2–4 μg mL^−1^ (Figure 4). Even at 8 μg mL^−1^ gentamicin, there was still detectable growth in one of the replicates when CFUs were measured (Figure 4B). In total, the monomicrobial checkerboard data revealed that CFU data are at least as reliable as the turbidity data. Additionally, CFU data enabled a differentiation between bactericidal and bacteriostatic antibiotic concentrations. Because the initial inoculum was ~3 × 10^6^ CFU mL^−1^ (Supplementary Table S1), the plating strategy outlined in the methods would yield a CFU count of ~3 colonies per well for the initial inoculum. Therefore, antibiotic concentrations resulting in no detectable growth would be considered bactericidal, conditions resulting in a low number of colonies (~1–10 CFU) could be considered bacteriostatic since they prevented further growth but did not kill the inoculum, and conditions resulting in numbers greater than 10–20 CFU should be assumed to support bacterial growth even in cases in which growth did not reach visually detectable turbidity.

In general, checkerboard assays demonstrate whether two drug interactions will provide a positive combined effect (synergistic) or a negative combined effect (antagonistic) [14]. For the purposes of this study, we are defining synergistic effects as combinations in which the MIC of one antibiotic decreases in the presence of another antibiotic. Antagonistic effects will be defined as combinations in which the MIC of one antibiotic increases in the presence of another antibiotic. Additionally, we will define instances in which the MIC of an antibiotic is unchanged by the presence of another antibiotic as “indifference”. The synergistic effect of the clinically used ceftazidime and gentamicin combination [8,9] is most strikingly observed in *P. aeruginosa* (Figure 4), which is not surprising considering that this combination is specifically recommended for treatment of *P. aeruginosa* infections. This synergistic interaction was confirmed using the calculations described above, resulting in a positive PVOA and PVOV of 30.56 and 30.73, respectively. For *S. aureus*, *E. faecalis*, and *A. baumannii*, the combination of ceftazidime and gentamicin displayed overall MIC indifference with only extremely subtle additive effects (Figure 1, Figure 2 and Figure 3).

**Figure 5:**
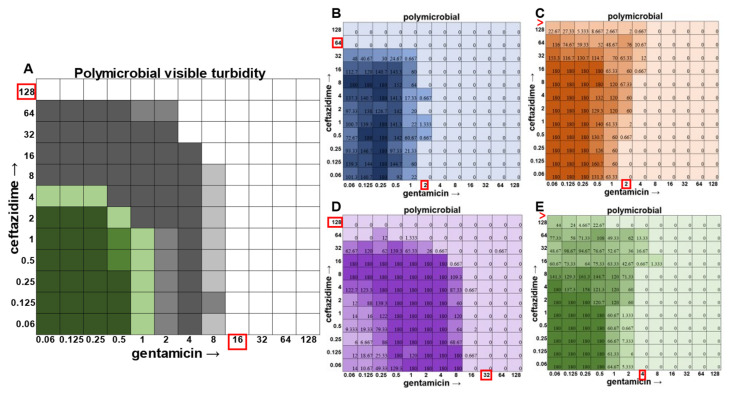
Checkerboard assay with the four species within the polymicrobial community. (**A**) Visible turbidity of the polymicrobial community consisting of all four species. Data represent triplicate checkerboard assays with ceftazidime and gentamicin. Green represents where *P. aeruginosa* pigmentation is present, while gray represents turbidity without green pigmentation. Dark coloration indicates that turbidity was visible in the well in all replicates, medium coloration indicates that turbidity was visible in two replicates, and light coloration indicates that turbidity was visible in one replicate. (**B**–**E**) CFU counts from each individual species when grown in the polymicrobial condition are shown on the right ((**B**) *S. aureus* in blue, (**C**) *E. faecalis* in orange, (**D**) *A. baumannii* in purple, and (**E**) *P. aeruginosa* in green). As shown above, bacteria are still present even after there is no longer any visible turbidity. The loss of *P. aeruginosa*’s green pigmentation is also associated with the loss of interspecies competition, as shown by the increase in *A. baumannii*’s population as the pigment disappears from view. Data represent averages of triplicates performed on different days. Red boxes indicate determined MIC for each species and the polymicrobial community. Red greater-than sign indicates MIC > 128.

Once the methodology for cell viability readouts had proved effective in monoculture checkerboard assays, we sought to assess the usefulness of this technique in the polymicrobial community. It should be noted that, in addition to turbidity, we observed that we could visualize *P. aeruginosa* growth via the visual detection of its green pigment pyocyanin (Figure 5A). However, the cellular source of the turbidity for all other members of the community was indistinguishable, necessitating the use of CFU plating on differential and selective media. Indeed, plating on Mannitol Salt Agar for *S. aureus* (Figure 5B), Bile Esculin Agar with Azide for *E. faecalis* (Figure 5C), Leeds for *A. baumannii* (Figure 5D), and Pseudomonas Isolation Agar for *P. aeruginosa* (Figure 5E) enabled MIC determination for each individual member of the community.

**Figure 6:**
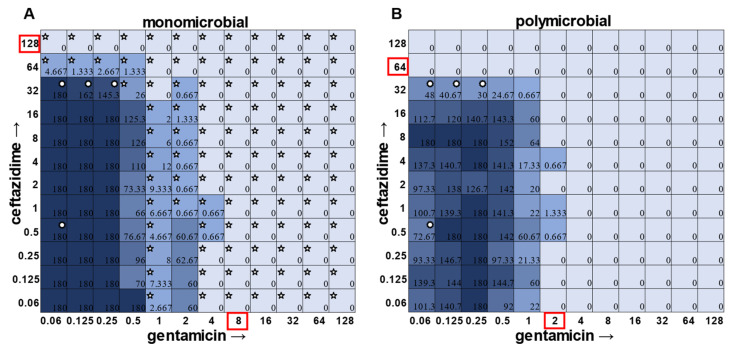
Monomicrobial versus polymicrobial checkerboards reveal that *S. aureus* is slightly sensitized to both ceftazidime and gentamicin in polymicrobial culture. CFU counts for *Staphylococcus aureus* ATCC 29213 when grown in the (**A**) monomicrobial condition versus the (**B**) polymicrobial condition. Red boxes indicate determined MIC. Five pointed stars in the top left corner of a well represent a CFU count that is significant compared to the growth control (180 for monomicrobial and 125.33 for polymicrobial). Circular stars in the top right corner of a well represent a CFU count that is statistically significant between monomicrobial and polymicrobial conditions (two-sample *t*-test).

**Figure 7:**
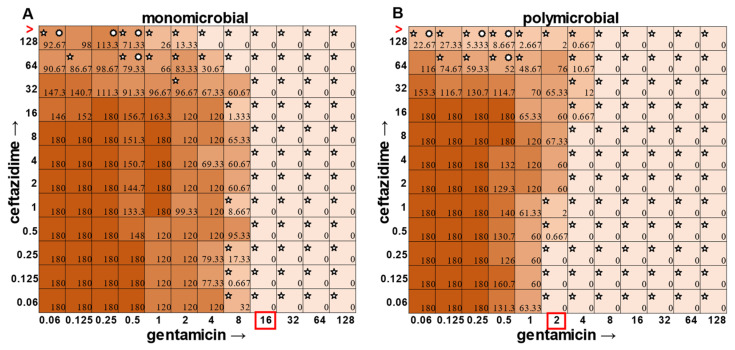
Monomicrobial versus polymicrobial checkerboards reveal that *E. faecalis* is sensitized to gentamicin in polymicrobial culture, but this effect is antagonized by ceftazidime. CFU counts for *E. faecalis* ATCC 29212 when grown in the (**A**) monomicrobial condition versus the (**B**) polymicrobial condition. Red boxes indicate determined MIC. Red greater-than sign indicates MIC > 128.5; pointed stars in the top left corner of a well represent a CFU count that is significant compared to the growth control (180 for monomicrobial and 180 for polymicrobial). Circular stars in the top right corner of a well represent a CFU count that is statistically significant between monomicrobial and polymicrobial conditions (two-sample *t*-test).

**Figure 8:**
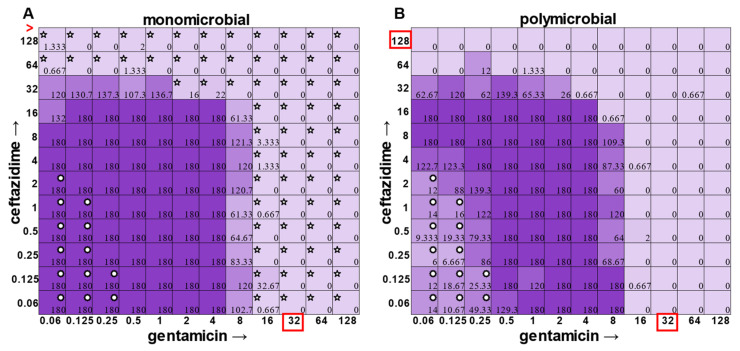
Monomicrobial versus polymicrobial checkerboards reveal that *A. baumannii* succumbs to interspecies competition with low levels of antibiotic, but overall, MICs are unchanged. CFU counts for *A. baumannii* ATCC 19606 when grown in the (**A**) monomicrobial condition versus the (**B**) polymicrobial condition. Red boxes indicate determined MIC. Red greater-than sign indicates MIC > 128. Five pointed stars in the top left corner of a well represent a CFU count that is significant compared to the growth control (180 for monomicrobial and 63.33 for polymicrobial). Circular stars in the top right corner of a well represent a CFU count that is statistically significant between monomicrobial and polymicrobial conditions (two-sample *t*-test).

In the polymicrobial community, we were able to observe some interspecies competitive behavior. For example, in wells in which *P. aeruginosa* was producing pyocyanin (Figure 5A), a growth suppression of *A. baumannii* was observed that was reversed when antibiotic concentrations were high enough to inhibit the production of virulence factors in *P. aeruginosa* (Figure 5D). It is worth noting that competition in polymicrobial communities can also make some individuals more susceptible to antibiotics. For example, *S. aureus* became sensitized to gentamicin at 2 μg mL^−1^ and ceftazidime at 64 μg mL^−1^ in the polymicrobial condition relative to monomicrobial culture, although overall, the combination of the antibiotics exhibited MIC indifference in either culture condition (Figure 6). Even more strikingly, *E. faecalis* became dramatically sensitized to gentamicin in the polymicrobial context, although the addition of increasing amounts of ceftazidime antagonized this effect (Figure 7). Interestingly, the antibiotic MIC for *A. baumannii* appears to be unaffected by the polymicrobial community, even though it was evident that its growth was likely inhibited by its polymicrobial competitor *P. aeruginosa* at lower antibiotic concentrations and the ceftazidime/gentamicin combination for *A. baumannii* displayed relative MIC indifference with only subtle additive effects in either condition (Figure 8). 

**Figure 9:**
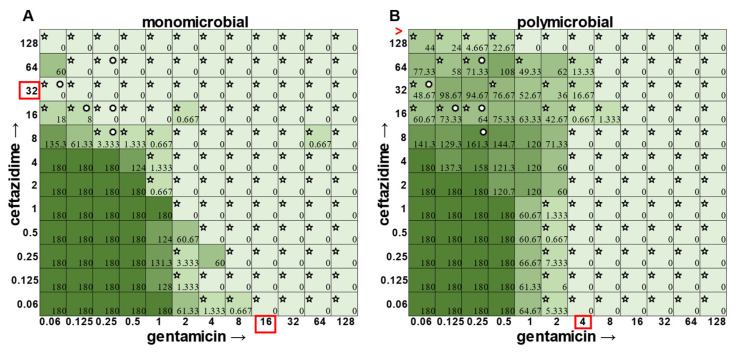
Monomicrobial versus polymicrobial checkerboards reveal that the additive effect of ceftazidime and gentamicin against *P. aeruginosa* becomes antagonistic in polymicrobial culture. CFU counts for *P. aeruginosa* ATCC 27853 when grown in the (**A**) monomicrobial condition versus the (**B**) polymicrobial condition. Red boxes indicate determined MIC. Red greater-than sign indicates MIC > 128.5. Pointed stars in the top left corner of a well represent a CFU count that is significant compared to the growth control (180 for monomicrobial and 180 for polymicrobial). Circular stars in the top right corner of a well represent a CFU count that is statistically significant between monomicrobial and polymicrobial conditions (two-sample *t*-test).

The most surprising finding of this study was that the gentamicin and ceftazidime combination shown to be effective against *P. aeruginosa* when grown in the monomicrobial condition (Figure 4 and Figure 9A) became highly antagonistic in polymicrobial culture (Figure 9B). Antibiotic interaction calculations confirmed the relationship between antibiotics to be antagonistic, with a negative PVOA and PVOV of −2.78 and −4.196, respectively. These negative PVOA and PVOV results indicating an antagonistic antibiotic interaction are in stark contrast to the positive PVOA and PVOV observed in the monomicrobial suspension indicating antibiotic synergy. This is concerning as hospitals base their antimicrobial susceptibility testing on monomicrobial suspensions, which in this case, would be counterproductive to effective patient treatment, as this combination appears to work well when *P. aeruginosa* is grown by itself but is no longer effective when *P. aeruginosa* is present in the community.

## 3. Discussion

As displayed in the results herein, current checkerboard methodology, which focuses on the determination of the MIC of two antibiotics using visible turbidity, cannot account for polymicrobial interactions leading to shifts in the antibiotic susceptibilities of individual species. These shifts can either be antagonistic or synergistic, depending on the antibiotic and the species within the community. This new method does not focus just on visible turbidity or optical density readings, but also accurate CFU counts to be determined for each species in a polymicrobial community, allowing for more accurate changes in individual susceptibilities to be determined. Many infections are polymicrobial, especially those that are chronic or persistent in nature [16,17,18], and it has been shown that an organism’s community can play a role in determining its antimicrobial susceptibility. As the community can both help (co-culture with *S. aureus* causes *P. aeruginosa* to alter its biofilm structure to make it resistant to tobramycin [19]) or be a hinderance (cooperative cross-feeding with anaerobic bacteria causing the now-dependent *P. aeruginosa* to become less resilient when challenged with antibiotics [20]), it is necessary to account for community dynamics when determining individual antimicrobial susceptibilities, to allow for better treatment of patient infections.

The determination of polymicrobial MICs is especially important in cases where the community renders current combinatorial therapies either useless or counterproductive. In the case of gentamicin and ceftazidime, a currently recommended therapy for *P. aeruginosa* [8,9], this combination becomes antagonistic when *P. aeruginosa* is present in a polymicrobial community. Antimicrobial susceptibility testing (AST) performed with the monomicrobial culture of *P. aeruginosa* reveals an MIC of 32 μg mL^−1^ of ceftazidime needed to completely clear the infection. However, when the community is present, the MIC of ceftazidime against *P. aeruginosa* jumps to >128 μg mL^−1^ (Figure 9). Additionally, if a patient with a polymicrobial infection containing *P. aeruginosa* was to be prescribed a combination of gentamicin and ceftazidime to treat their chronic infection with the dosage based on monomicrobial MICs determined via visible turbidity, our data show that the addition of higher concentrations of ceftazidime could reverse the usefulness of gentamicin (Figure 9B). As the effectiveness of treatment influences both patient morbidity and mortality, we propose that it is crucial to consider the role of a pathogen’s community when determining the best treatment. Future directions for this project include the determination of essential community members and the role that they play in protecting *P. aeruginosa* from the combinatorial therapy of gentamicin and ceftazidime, as well as testing clinical isolates using this new methodology.

**Figure 10:**
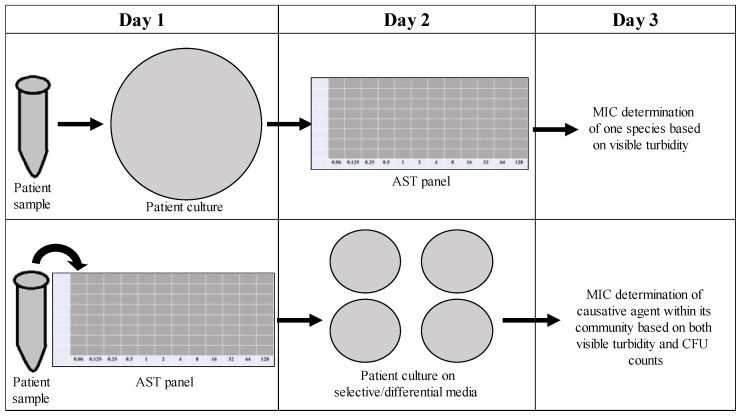
New checkerboard methodology allows for determination of CFUs in a polymicrobial community without extending time needed to determine antibiotic susceptibilities. This represents a comparison of the current method of antimicrobial susceptibility testing (AST) (**top** row) versus our proposed AST methods (**bottom** row). As shown in the figure, our method does not extend testing time, and yet allows for a more accurate determination of the MIC of antimicrobials used to treat the causative agent. This method not only accounts for the role of the community in determining MIC, but also allows for CFU counts to be obtained, providing an accurate depiction of growth even when visible turbidity shows none.

As time is often a concern with treating persistent infections (the longer an infection lasts, the more at risk a patient becomes for complications), it would be undesirable to implement new techniques in the clinical microbiology laboratory that require additional time. Our new method for determining the CFU counts of the individuals in a polymicrobial community focuses on effectively determining the role of the community in influencing antimicrobial susceptibility within the same time frame as one might determine the MIC of a monomicrobial AST panel. As shown in Figure 10, instead of overnight culturing to determine the causative agent of infection in pure culture microbiology, patient samples could be placed directly into an AST panel and plated after 18 h on selective/differential media to visualize the different members of the community contributing to changes in antimicrobial susceptibilities, as well as the one causative agent of infection. Samples collected from patients with excess turbidity due to blood or sputum could be filtered to remove unwanted cells or other nonbacterial components. This new methodology would not significantly increase the time needed for MIC determination and would allow for better analysis of how the community affects individual antibiotic susceptibilities, therefore leading to the better determination of MICs and more effective prescription of treatment. 

## 4. Materials and Methods

### 4.1. Bacterial Isolates and Media

The four isolates used in these experiments were *S. aureus* ATCC^®^ 29213, *P. aeruginosa* ATCC^®^ 27853, *E. faecalis* ATCC^®^ 29212, and *A. baumannii* ATCC^®^ 19606. *S. aureus* grew yellow colonies on Mannitol Salt Agar (Fisher Scientific™, Hampton, New Hampshire). *P. aeruginosa* grew green colonies on *Pseudomonas* Isolation Agar (Fisher Scientific™). *E. faecalis* grew black colonies on Bile Esculin Agar with Azide (VWR^®^, Radnor Pennsylvania). *A. baumannii* grew pink colonies on Leeds (VWR^®^).

### 4.2. Preparation of Microbial Overnight Cultures

A sterile loop was used to obtain approximately five colonies from a selective/differential plate. The colonies were then inoculated into a 125 mL glass flask containing 5 mL of LB and incubated for 18 h in a shaking incubator at 200 rpm and 37 °C under ambient air conditions. After 18 h of incubation, the overnight cultures contained approximately 10^9^ CFU mL^−1^ as shown by diluting and plating on the appropriate selective/differential media for each species.

### 4.3. Antibiotic Preparation and Dilution

All wells in a 96-well plate were filled with 100 μL of Cation-Adjusted Mueller Hinton broth (CAMHB). A 256 μg mL^−1^ storage stock was created for each antibiotic, using methods from the Clinical Laboratory and Standards Institute (CLSI) manual [21,22]. Briefly, 100 μL of 256 μg mL^−1^ concentration antibiotic stock for antibiotic A was added to all of the wells in the first column of the 96-well plate. A total of 100 μL from that first column was then diluted using serial dilutions across the rest of the wells, all the way to column 11 (1:2 dilutions starting with a concentration of 128 μg mL^−1^ and ending with a final concentration of 0.125 μg mL^−1^). This process was then repeated in a separate 96-well plate for antibiotic B (Supplementary Figure S1).

### 4.4. Preparation of Microbial Inoculum

A spectrophotometer was used to measure the OD_625_ of each bacterial species as per the CLSI protocol [21,22]. The bacteria were then diluted in 1X PBS to a McFarland standard of 0.08. Next, 40 μL of the McFarland standard was added to 760 μL 1X PBS to create the inoculums for each species. For the polymicrobial inoculum, the 40 μL was split evenly between the 4 species (10 μL per species). Inoculum CFU mL^−1^ obtained from plating on selective/differential media was reported (Supplementary Table S1).

### 4.5. Checkerboard Assay Setup

In two empty 96-well plates, 45 μL of each antibiotic was added at one concentration higher than desired, with one antibiotic in columns, and one in rows (for example, when a concentration of 0.25 μg mL^−1^ of gentamicin was desired, 45 μL of 0.5 μg mL^−1^ gentamicin would be added to the well in addition to 45 μL of the other desired concentration of ceftazidime) (Supplementary Figure S2). The 128 μg mL^−1^ wells were filled from the 256 μg mL^−1^ antibiotic stocks. Each species had its own checkerboard, and the community was given its own checkerboard (Supplementary Figure S3). At the edge of one of the panels, five wells were filled for the growth control (one per species and one for the community), five wells were filled for the vehicle control (sodium carbonate plus water for ceftazidime), and two wells were filled for the contamination check (one with the vehicle and one without). All wells except for the contamination check wells were inoculated with 10 μL of the appropriate microbial inoculums, and the checkerboards were then incubated for 18 h. At this time, each inoculum was diluted out ranging from 10^−1^ to 10^−8^ in 1X PBS and then 5 μL of each dilution was plated on selective/differential media. The inoculum plates were incubated along with the panels to obtain the CFU mL^−1^ of the inoculum.

### 4.6. Preparation of 96-Well Microplates for Dilutions

A sterile pipetting reservoir was filled with 1X PBS. For the 1:10 dilution (first dilution), 90 μL of 1X PBS was added to each well of a 96-well plate. For the 1:100 dilution (second dilution), 198 μL of 1X PBS was added to each well in a 96-well plate.

### 4.7. Diluting and Plating the Checkerboard

After 18 h, the checkerboards were read for visible turbidity. All wells were then diluted twice by pipetting 10 μL into 90 μL (1:10 dilution), followed by 2 μL into 198 μL (1:100 dilution) for a total dilution factor of 1:1000. Then, 5 μL of each of these dilutions was plated on selective and differential media (Supplementary Figure S4). After incubation for 18–24 h, colony counts were obtained for each species in both the polymicrobial and monomicrobial conditions at each concentration for each antibiotic represented in the checkerboards. CFU counts were multiplied by two to be representative of plating 10 μL and were reported as CFU mL^−1^.

### 4.8. Data Analysis

All doubled CFU counts were recorded in an Excel spreadsheet representing the checkerboard wells. Biological repeats were averaged together to obtain the average CFU for each well (180 CFU was substituted for wells that were “TNTC” (“too numerous to count”) as an upper limit on CFU counts to enable data averages to be determined among replicates). Conditional formatting was used in Excel to display the varying CFU counts for each well (the higher the CFU count, the darker the color of the well). All data present in the graphs are representative of the average of three replicates. A two-sample *t*-test was performed to compare both monomicrobial and polymicrobial data to their respective growth controls for each species, as well as to compare monomicrobial versus polymicrobial CFU counts. *p*-values < 0.05 were reported as significant.

### 4.9. Calculating Antibiotic Synergy or Antagonism

As a measure of the efficacy of given antimicrobial combinations, we proposed two methods comparing the predicted indifference in two antimicrobial drugs with the observed interaction between the drugs: (1) predicted indifference area vs. observed interaction area (PVOA), and (2) predicted indifference volume vs. observed interaction volume (PVOV). The first method used only visible turbidity (for example, when CFU counts were not available). The second method used available CFU counts and gave a perhaps more nuanced interpretation of the checkerboard data.

In the first method, when the CFU counts were not available, we calculated the predicted indifference area vs. the observed interaction area (PVOA). We first defined the “predicted indifference area” (PA) and “observed interaction area” (OA) of a 12 × 12 checkerboard assay. The predicted indifference area is simply the area of the rectangular region defined by the indifference lines for gentamicin and ceftazidime (with each grid square viewed as having a unit area). Similarly, the observed interaction area (OA) is the area of all of the grid squares showing visible turbidity. We could then calculate PVOA, which we defined to be the percent change between PA and OA. This value gave us a measure of synergistic or antagonistic interaction. The PVOA (percent change between PA and OA) is given by

$$\mathrm{PVOA}=100\times \left(\mathrm{PA}\;-\;\mathrm{OA}\right)/\mathrm{PA}.$$

Positive PVOA means that the observed area of turbidity is smaller than the area predicted by the indifference lines. This represents synergistic interaction. Similarly, negative PVOA means that the observed area of turbidity is greater than the area predicted by the indifference lines, which indicates antagonistic interaction.

To calculate predicted indifference volume vs. observed interaction volume (PVOV), we first define “predicted indifference volume” (PV) and “observed interaction volume”(OV) of a given checkerboard assay. Given a 12 × 12 checkerboard assay with CFU counts, we view the assay results as a 12 × 12 grid of vertical columns (a 3D histogram), where each column has a unit square base with the column height given by the CFU count. Thus, the volume of one column in the 3D histogram is equal to the CFU count in that square (1 × 1 × CFU). The total “volume” of the assay, the “observed interaction volume” (OV), is then the sum of all CFU counts in the checkerboard (i.e., the sum of all 3D histogram columns). For comparison, we define the “predicted indifference volume” (PV) to be the volume of the region enclosed by the indifference lines for gentamicin and ceftazidime. For the height of each column in this region, we use the mean of the nonzero CFU counts in the assay. Therefore, the predicted indifference volume (PV) is simply the predicted indifference area (PA) multiplied by the mean of the nonzero CFU counts. Once we have a predicted indifference volume (PV) and observed interaction volume (OV), we calculate the PVOV, which we define to be the percent change between PV and OV. This value gives us a measure of synergistic or antagonistic interaction. The PVOV (percent change between PV and OV) is given by

$$\mathrm{PVOV}=100\times \left(\mathrm{PV}\;-\;\mathrm{OV}\right)/\mathrm{PV}.$$

Positive PVOV means that the observed volume of turbidity is smaller than the volume predicted by the indifference lines. This represents synergistic interaction. Similarly, negative PVOV means that the observed volume of turbidity is greater than the volume predicted by the indifference lines, which indicates antagonistic interaction. A sample calculation can be found in Supplementary Figure S5.

## Data Availability

Data is contained within the article or Supplementary Material.

## Supplementary Materials

The following supporting information can be downloaded at: https://www.mdpi.com/article/10.3390/antibiotics12071207/s1, Figure S1: Methods for diluting both antimicrobials start with a 256 μg mL^−1^ stock, followed by 1:2 dilutions to a concentration of 0.125 μg mL^−1^; Figure S2: Checkerboard setup has one antimicrobial in rows and one antimicrobial in columns, both with varying concentrations; Figure S3: The completed checkerboard setup for these experiments consisted of eight 96-well plates with the four species and the community split between them; Figure S4: CFUs were obtained by plating 5 μL onto selective/differential media, and then incubating them for 18–24 h; Figure S5: No discernable differences between observed visible turbidity and optical density (OD) readings were detected; Figure S6: Calculations of PVOA and PVOV confirm that the combination of gentamicin and ceftazidime is antagonistic for *P. aeruginosa* present in the polymicrobial community as compared to synergistic, when tested alone; Table S1: Inoculum CFU mL^−1^ obtained for each species shows inoculums were very similar across species in the polymicrobial condition.
