# Supplementary material for: Development of a Polymicrobial Checkerboard Assay as a Tool for Determining Combinatorial Antibiotic Effectiveness in Polymicrobial Communities
Source: Antibiotics (Basel). 2023 Jul 20;12(7):1207. doi: 10.3390/antibiotics12071207 (PMC10376321; doi:10.3390/antibiotics12071207)
Supplement: Supplementary file 1 [file antibiotics-12-01207-s001.zip › antibiotics-2452840-supplementary.pdf]

antibiotics

MDPI

Supplementary Materials:

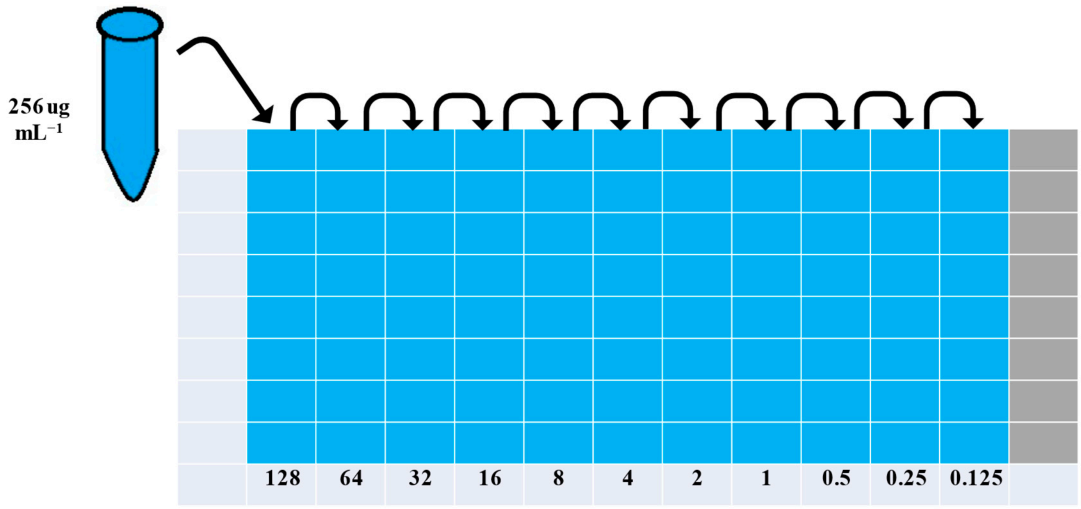

Supplementary Figure S1. Methods for diluting both antimicrobials start with a 256 μg mL^{-1} stock, followed by 1:2 dilutions to a concentration of 0.125 μg mL^{-1}**.** Depiction of serial dilutions performed for both antimicrobials. A total of 100 μL of a 256 μg mL^{-1} stock was diluted out in 100 μL of CAMHB (1:2 dilutions) to a final concentration of 0.125 μg mL^{-1}; 45 μL of each dilution was then added to the checkerboard itself.

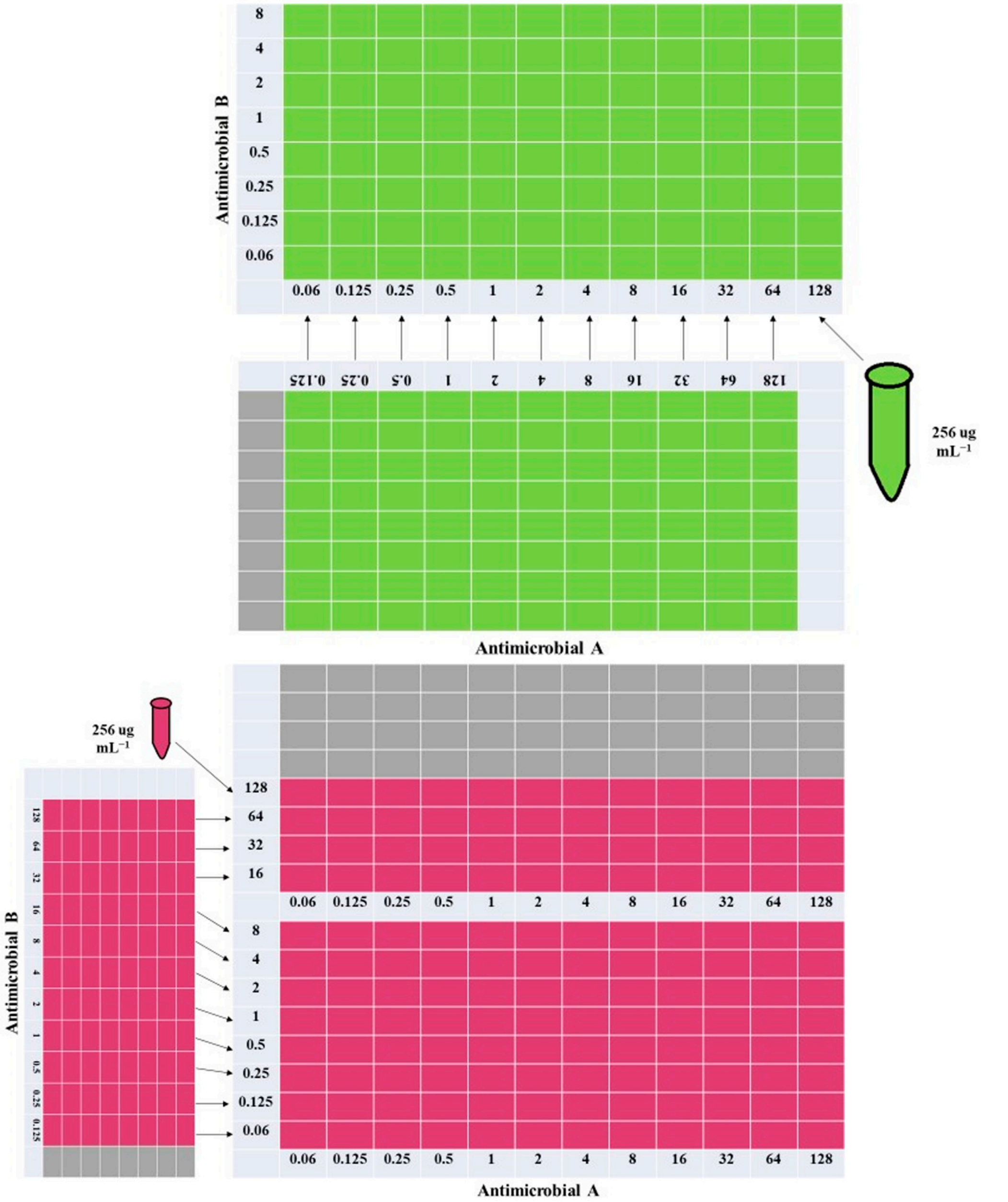

Supplementary Figure S2. Checkerboard setup has one antimicrobial in rows and one antimicrobial in columns, both with varying concentrations**.** Each well in the checkerboard received 45 µL of each antimicrobial from a well, one concentration higher than desired. For example, if a concentration of 4 µg mL $^{-1}$ was desired, 45 µL would be taken from the 8 µg mL $^{-1}$ well.

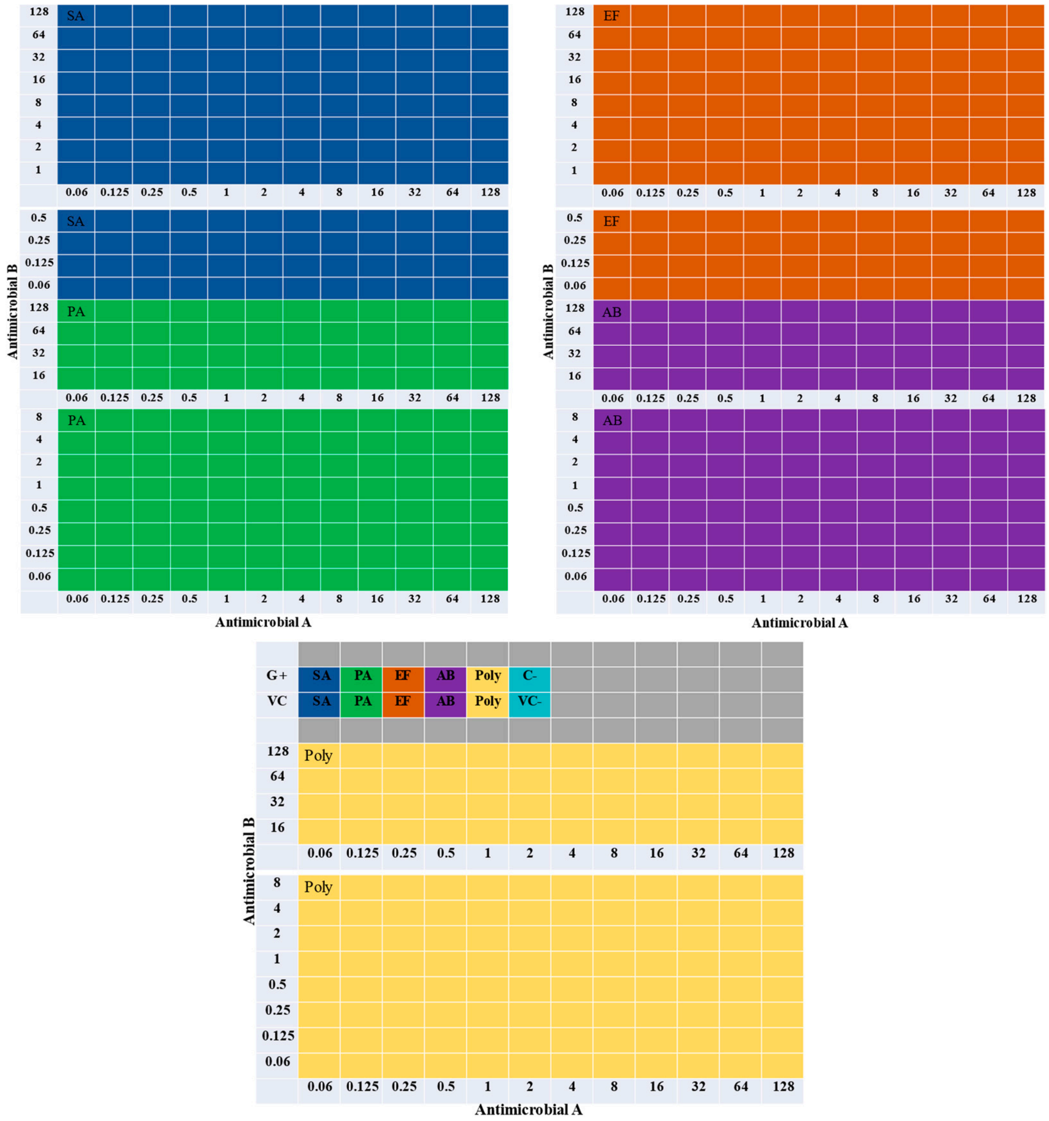

Supplementary Figure S3. The completed checkerboard setup for these experiments consisted of eight 96-well plates with the four species and the community split between them. Depiction of the complete checkerboard shows that each species had 144 wells of antibiotic split across two 96-well plates, a growth control well, and a vehicle control well. A contamination check with and without the vehicle was also present on another 96-well plate.

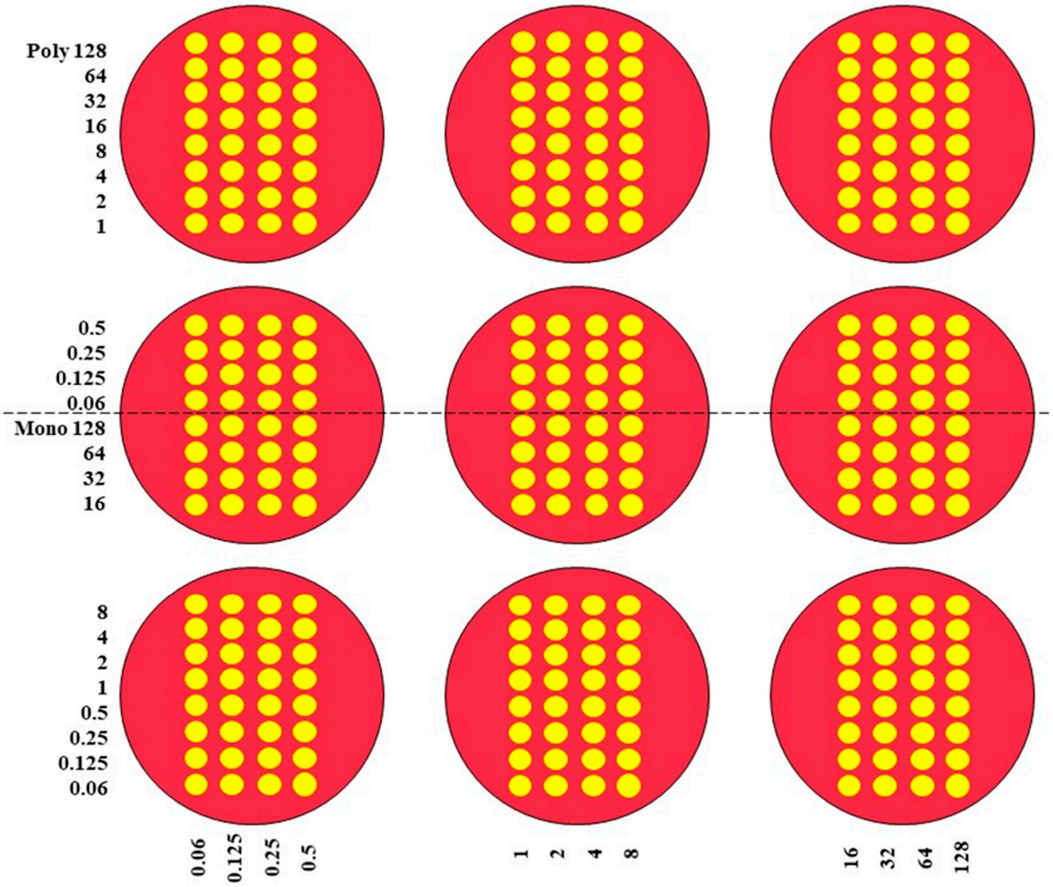

Supplementary Figure S4. CFUs were obtained by plating 5 μL onto selective/differential media, and then incubating them for 18–24 h. Depiction of plate setup. A total of 5 μL plated onto selective/differential media. The above setup existed for each individual species. The growth control and vehicle control were plated at the edge of a plate. CFU counts were obtained after incubation by multiplying by two to represent what would have been obtained if 10 μL had been plated.

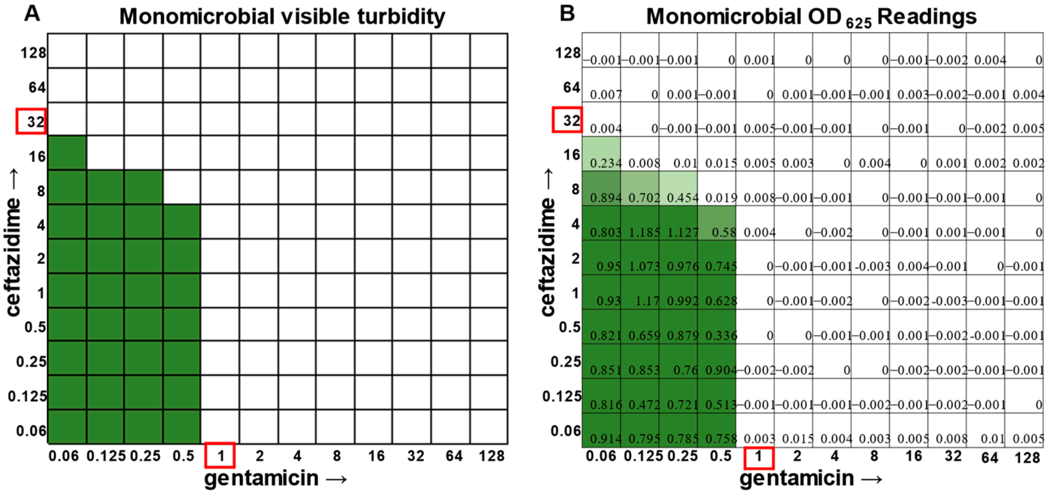

Supplementary Figure S5. No discernable differences between observed visible turbidity and optical density (OD) readings were detected. OD_{625} readings were taken for each species and the community after 18 h incubation with no discernable differences observed between visible turbidity detected by the human eye and OD_{625} readings. The figure above shows data representative of this phenomenon, collected from monomicrobial *P. aeruginosa*. OD_{625} values were standardized to 0.04 to account for background subtraction.

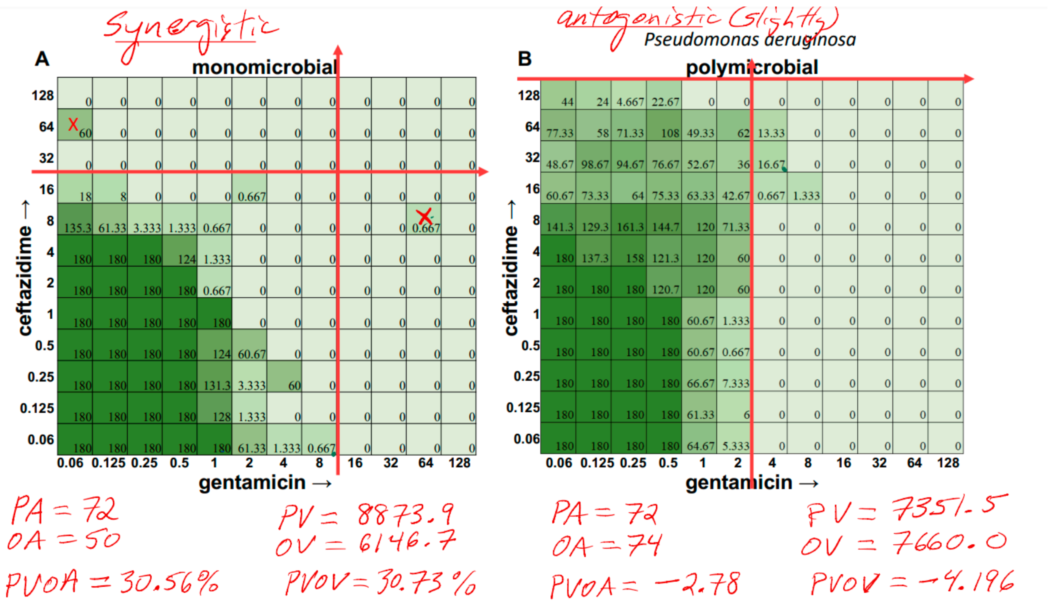

Supplementary Figure S6. Calculations of PVOA and PVOV confirm that the combination of gentamicin and ceftazidime is antagonistic for *P. aeruginosa* present in the polymicrobial community as compared to synergistic, when tested alone. Using the calculations described in the methodology, it was determined that while ceftazidime and gentamicin appear to be a synergistic combination when tested against *P. aeruginosa* monomicrobially (with a PVOA and PVOV of 30.56 and 30.73, respectively), when tested against *P. aeruginosa* in the polymicrobial condition, the same antibiotic combination was revealed to be antagonistic with a PVOA and a PVOV of −2.78 and −4.196, respectively.

Supplementary Table S1. Inoculum CFU mL^{-1} obtained for each species shows inoculums were very similar across species in the polymicrobial condition. Average CFU mL^{-1} for each species' inoculum for both the monomicrobial and polymicrobial conditions. The standard deviation is also included.

Species	Mono Avg. CFU/mL	Mono St. Dev.	Poly Avg. CFU/mL	Poly St. Dev.
SA 29213	4.28 x 10^6	8.35 x 10^5	1.38 x 10^6	2.71 x 10^5
PA 27853	8.44 x 10^6	4.10 x 10^6	1.80 x 10^6	4.24 x 10^5
EF 29212	6.22 x 10^5	4.85 x 10^5	8.67 x 10^5	1.63 x 10^5
AB 19606	4.97 x 10^6	7.52 x 10^5	2.07 x 10^6	9.49 x 10^5
